# Correction: Min et al. Korean Version of the Nursing Student Attitudes and Knowledge toward Lesbian, Gay, Bisexual, and Transgender Patients Scale. *Healthcare* 2023, *11*, 2028

**DOI:** 10.3390/healthcare13070754

**Published:** 2025-03-28

**Authors:** Hye-Young Min, Jungmin Lee, James Montegrico, Hee-Jung Jang

**Affiliations:** 1 College of Nursing, Ewha Womans University, Seoul 03760, Republic of Korea; hyeyoungmin@ewhain.net; 2 School of Nursing, Hallym University, Chun-cheon 24252, Republic of Korea; j_lee0624@hallym.ac.kr; 3 School of Nursing, University of North Carolina at Charlotte, Charlotte, NC 28223, USA; jmontegr@uncc.edu

Adapting and translating tools for use in different cultures involves multiple approaches. When terms are directly translated from one language to another, they can sometimes seem odd due to differences in language and culture. Instead of creating something entirely new, adaptation typically involves tweaking an existing tool to fit the cultural specifics of a new audience. This process maintains the original effectiveness of the tool while ensuring that it resonates and functions well in its new cultural context.

The authors would like to make the following corrections to the published paper [1]. The changes are as follows:

**Text Correction**

Describing data as a development process can be misleading. “The instruments used for assessing reliability and validity in this study were applied cross-culturally and were originally developed by Cornelius and Carrick [14].” (Original statement: The LGBT health concern knowledge and attitude measurement tool [16] was developed to measure the knowledge that nursing college students possess regarding various areas of LGBT individual healthcare (e.g., cancer risk, cardiovascular risk, vaccination, human immunodeficiency virus infection and acquired immune deficiency syndrome, sexually transmitted diseases, drug abuse, sex reassignment surgery, smoking, and violence).)

The following correction has been made to the Introduction, paragraph 5:

“In the future, we will objectively evaluate the level of knowledge and heterosexual attitudes of Korean nursing college students using the scale cross-culturally translated and adapted in this study, accumulating basic data for the development of LGBT nursing education programs in South Korea.”

The following correction has been made to the Discussion, paragraph 1:

“The adaptation of the NKALH Scale adds to the literature by providing a culturally appropriate instrument for measuring the attitudes and knowledge of Korean nursing students regarding LGBT individuals.”

Additionally, we want to provide details regarding the backward–forward translation process, such as who performed the translation, the translators’ experience with the translated questionnaire, and who assessed the quality of the translation, which are key to establishing validity of the cultural adaptation process.

The following correction has been made to the Materials and Methods, *Study Process*, Translation Process, paragraph 1:

“We followed the cross-cultural adaptation guidelines outlined by the World Health Organization [17] for our translation process, which included semantic translation. Initially, two independent translators, both native Korean speakers with proficiency in English, carried out the forward translation. The co-author was involved in the forward translation, along with a professor from the School of Nursing in South Korea, who were not affiliated with our study, participated in the forward translation process. Both forward translators possessed degrees or had been visiting scholars in the USA, ensuring their linguistic competence.

Following the forward translation, an expert panel conducted a comprehensive review to identify and resolve any inconsistencies between the original English version and the translated Korean version. This panel consisted of three experts, including the two experts from the forward translation phase. Another panel member was a professor from the School of Nursing in South Korea, who is an expert in risky sexual behavior.

Subsequently, two independent translators, fluent in both English and Korean and currently employed as nurses in the USA, conducted backward translation of the tool. To ensure the quality of the translation, all five experts collectively compared and deliberated on the English and Korean versions, resulting in the development of the pre-final Korean version of the tool.

Finally, four experts (excluding the co-author) rated each item on the Korean version, leading to the calculation of a content validity index (CVI) of 1.0, indicating unanimous consent agreement on all items within the Korean version of the NKALH.”

**References Correction**

With these corrections, the citation of some references has been adjusted accordingly.

The citation has now been corrected in Materials and Methods, *Measurement*, The LGBT Health Concern Knowledge and Attitude Measurement Tool, paragraph 1 and should be changed as follows:

“The Nursing Students’ Knowledge of and Attitudes Toward LGBT Healthcare [14] was developed to measure the knowledge that nursing college students possess regarding various areas of LGBT individual healthcare (e.g., cancer risk, cardiovascular risk, vaccination, human immunodeficiency virus infection and acquired immune deficiency syndrome, sexually transmitted diseases, drug abuse, sex reassignment surgery, smoking, and violence).”

The authors state that the scientific conclusions are unaffected. These corrections were approved by the Academic Editor. The original publication has also been updated.
